# CT definition of the surgical apex in the orbit

**DOI:** 10.1038/s41598-021-90419-9

**Published:** 2021-05-26

**Authors:** Olga Zurinam, Christine Safieh, Yael Redler, Adi Orbach, Dmitry Lumelsky, Ziv Neeman, Daniel Briscoe

**Affiliations:** 1 Department of Ophthalmology, Emek Medical Center, Afula, Israel; 2 Department of Radiology, Emek Medical Center, Afula, Israel; 3 Technion – Israel Institute of Technology, Haifa, Israel

**Keywords:** Anatomy, Diseases, Health care, Health occupations, Medical research, Neurology, Oncology, Risk factors

## Abstract

The orbital apex is an undefined but well understood concept of Orbital Surgeons. We sought to determine the surgical apex area specifically where the volume ratio decreases significantly impacting on the optic nerve. A retrospective analysis using PACS program processing, measured the right retrobulbar space volume changes in 100 randomly selected cases without orbital pathology where CT was performed for non-ophthalmic indications. Volume of the retrobulbar space was measured between two recognizable landmarks. The first landmark being the point of exit of the optic nerve from the eye and the second landmark the optic nerve's point of exit from the orbit. The measured length between these two points was divided into five equal segments, V1-V5. The volumes of all 5 segments were compared and the most significant area of volume depletion was established. The mean numeric value of measured orbital volumes was compared. A ratio difference of V1/V2 was less than 2, V2/V3 was 2.32 (± 0.27), V3/4 was 3.24 (± 0.39), and V4/V5 was 5.67 (± 1.66). The most remarkable difference in ratio was between V4 and V5 (mean 5.67 ± 1.66 with p < .0001). The V3 segment (the posterior 3/5 of the retrobulbar space volume) is the location where decrease in orbital volume impacts, and measured ratios are statistically significant. We defined the surgical apex as the posterior 3/5 of the retro-bulbar orbital space. It is consequently the area of higher risk for optic nerve compression. This definition could be routinely utilized by ophthalmologists and neuroradiologists when evaluating masses affecting the orbit.

## Introduction

The shape of the orbit is a pyramid with an anterior open base and a posterior apex^1,2^. Diseases affecting the orbit commonly involve retrobulbar area which contains a complex of muscles, nerves and blood vessels^3–5^. As we move more posteriorly towards the apex, the area becomes more confined and thus more difficult to approach surgically^6^. Sub-periosteal abscess, Thyroid associated eye disease, space occupying lesions, or trauma with bleeding can cause optic nerve compression in this area due to crowding and these problems often need surgical intervention^2,7–10^.

Most experienced orbital surgeons understand the concept of the orbital apex as a crowded and complex space demanding special attention. This conceptual space, "the surgical apex" has not been scientifically defined but is accepted as the posterior area of the orbit where the most significant decrease in volume occurs^11–13^. For example, an expanding sub-periosteal abscess when it reaches this area should theoretically pose a greater risk of optic nerve compression demanding urgent surgical attention. Decompression surgery of the orbit in Thyroid Associated Eye Disease due to optic nerve compression should theoretically address this area in particular. Defining where this critical area begins, as we go deeper into the orbit, might also help in anticipating difficulties in orbital biopsy.

In this study we used CT images of normal orbits and determined the area where volume depletion changes significantly as we move deeper into the orbital cavity. In this way we were able to define where the surgical apex is located.

## Materials and methods

A retrospective analysis of CT images, measuring right retrobulbar volume was carried out in a random sample of 100 patients without orbital pathology, from the database of CT scans performed at Emek Medical Center (EMC) between 2010–2013. Data included age, gender, race, and indication for CT.

All experimental protocol were approved by the EMC Ethics Committee review board. We confirm that study number for this study was 0045-11EMC. Methods were carried out in accordance with ICH-GCP and Ministry of Health guidelines, approved by the EMC Ethics Committee review board.

Waiver of informed consent was permitted by EMC Ethics Committee review board as the research was retrospective and anonymous presenting no threat to the rights and welfare of research subjects. The information is not sensitive in nature, and the data are derived from the clinically indicated CT Imaging already performed.

CT was performed for non-ophthalmological medical indications without any connection to our research (Table 2).

Using abnormal cases with orbital pathology would not have been appropriate in this study as the pathology could change the structure of the orbit. We therefore selected cases who had undergone high quality CT scans with thin slices (1 mm or less) for non-orbital indications excluding skull or orbital pathologies. High quality CT scans with thin slices were required as these could then be processed by the Picture Archive and Communication System (PACS) program for three-dimensional reconstructions of the orbit.

**Figure 1:**
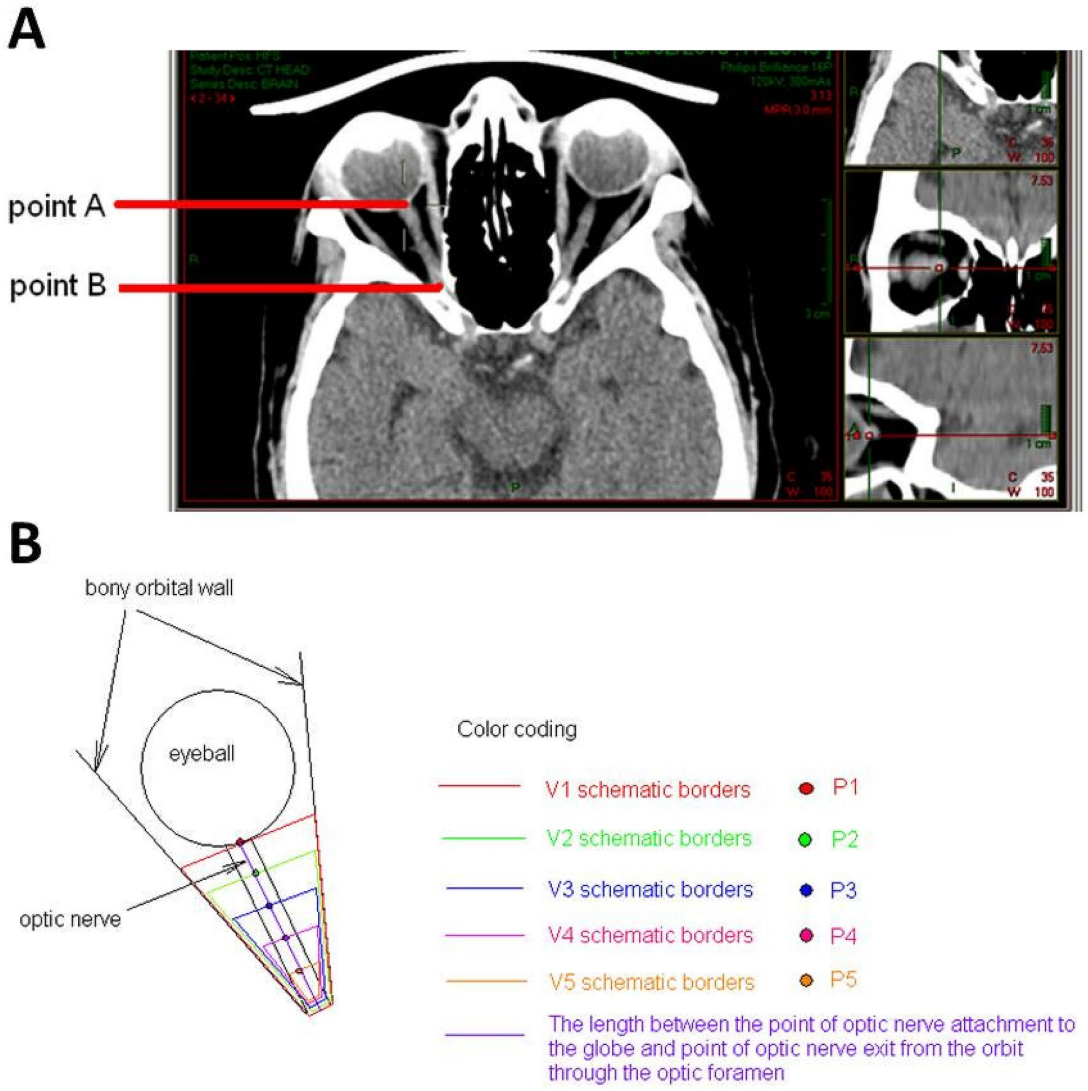
Anatomy and measurements. (**A**) CT scans of orbits in an axial plane with visualization of the intraorbital part of the optic nerve. Point A- the optic nerve exit point at the posterior part of the eyeball. Point B- the optic nerve entry to the optic foramen. (**B**) The measured length was divided into five equal segments designated by points P1to P5 . The total volume V1 was calculated by including all 5 segments, from P1 to the optic foramen. Four additional volume measurements were calculated by subtracting the anterior segment of the previously measured volume. V2 measured the area between P2, and the optic foramen measuring volume at 4/5 of total height V3, V4, andV5 measured volume at 3/5, 2/5,and 1/5 of total height respectively.

Retrobulbar volume was measured between two recognizable landmarks, the location of optic nerve—globe attachment and the exit point of the optic nerve from the orbit at the optic foramen. The exit point of the optic nerve from the orbit at the optic foramen was determined in an axial view, sliced at a height where the anterior clinoid processes could be identified as a landmark. Using the CT PACS program, we set constructed axial cross sections of the right orbit allowing good visibility of the entire length of the optic nerve between the two landmarks, points A and B (Fig. 1A).

The measured length was divided into five equal segments designated by points P1-P5. The total volume V1 was calculated by including all 5 segments, from P1 to the optic foramen. Four additional volume measurements were calculated by subtracting the anterior segment of the previously measured volume (Fig. 1B). We found that using fifths gave the best indication of volume change while utilizing the minimal number of sections possible. Using more sections would complicate the purpose of this study to define a general area which can be referred to as the surgical apex. Using thirds as often adopted by orbital surgeons would not be precise enough as volume reduction occurs suddenly as you move posteriorly.

V1–5 were calculated, compared, and statistically analyzed. The most significant area of volume depletion indicated the surgical orbital apex location.

**Figure 2:**
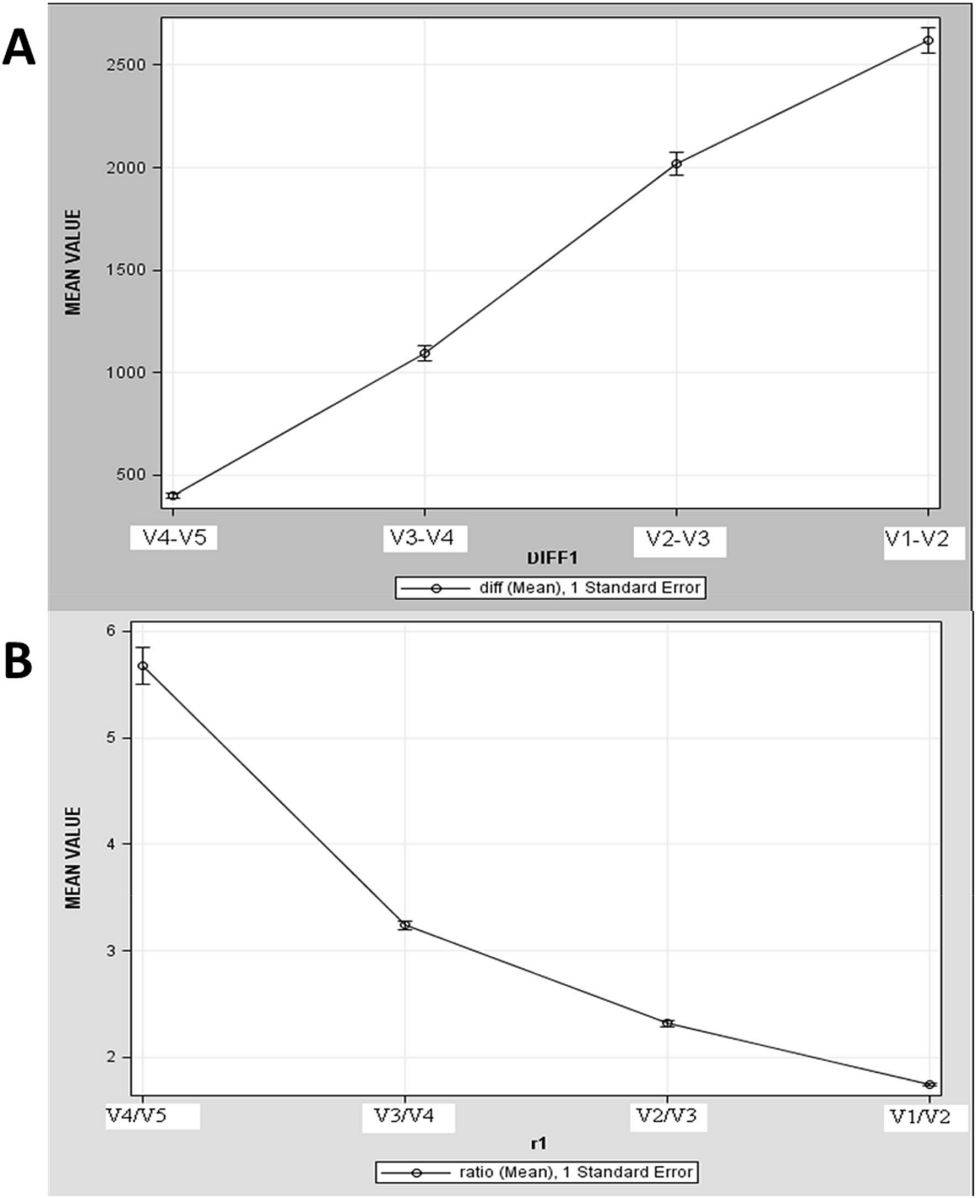
Orbital apex calculations and comparison. (**A**) The subtraction of differences in orbital volumes. The X axis – represents points of calculated differences between retrobulbar orbital volumes, they are shown at an equal distance from each other. The Y axis—represents the subtraction differences in nearest retrobulbar orbital volumes in mm^3^. Progression of a decline is most obvious between retrobulbar orbital volumes V2-V3 and V3-V4. (**B**) The X axis – represents points of calculated ratio differences between retrobulbar orbital volumes, they are shown at an equal distance from each other. The Y axis—represents ratio differences in the nearest retrobulbar orbital volumes. ProgressionSteeper progression of a decline is most obvious between V4/V3 and the most dramatic decline in the volumes occursis observed at the ratio between V4/V3 and V4/V5.

Retrobulbar volumes were compared with total patient data in two ways: subtraction of the smaller volume from the larger one (e.g. V1–V2), and division/ratio of the larger volume to a smaller one (e.g. V1/V2) (Fig. 2A,B).

## Results

100 CT scans of randomly selected patients performed at EMC, Afula—Israel from 01/01/2010 till 01/09/2013, were reviewed in accordance with the inclusion and exclusion criteria. The study was approved by the hospital Ethics committee and Scans were chosen in a blind random manner without knowledge the patient's gender, age, or ethnicity.

Statistical analysis was performed using SAS 9.2 (SAS Institute Inc., Cary, NC, USA). Categorical variables (such as gender, ethnicity etc.) are presented using frequencies and percentages. Continuous variables (such as age, volumes etc.) are presented using standard distribution measures (mean, standard deviation, range).

Repeated measures analysis was performed to explore changes along the differences between retrobulbar space orbital volumes. Significance was considered if P < 0.05.

Statistical analysis showed almost an equal number of male and female patients (52 men and 48 women) included in the study (Table 1). 94 CT scans were of adult patients and 6 were of children under the age of 10. Children under age 10 years old, had retrobulbar orbital volume measurements different from older participants. This small group of six is not enough to make statistically significant conclusions but does show a different trend. The mean age of the adult patients was 48.96 and the mean age of children under ten was 2.03. Almost all patients (99 out of 100) were Caucasian (Table 1).

**Table 1:** Participants Demographics by Gender and Age.

Characteristic		Number of patients	%
Gender	Male	52	52
	Female	48	48
Age	> 10 Years old	94	94
	< 10 Years old	6	6
Mean age	> 10 Years old	48.96	
	< 10 Years old	2.03	

The indications for performing the CT scan are shown in Table 2. The most frequent indications were trauma (56.38%), suspected space occupying lesion (8.51%), suspected cerebrovascular accident (7.45%), and headache (7.45%).

**Table 2:** Indication for CT examination in all patients.

Indication for CT	Frequency	%
Trauma	53	56.38
Suspected SOL	8	8.51
Suspected CVA	7	7.45
Headache	7	7.45
Otitis media	3	3.19
Dizziness	3	3.19
Suspected sinusitis	2	2.13
Recurrent falls	2	2.13
Dementia	1	1.06
Foot drop	1	1.06
Loss of consciousness	1	1.06
Fainting	1	1.06
General weakness	1	1.06
Hearing loss	1	1.06
Diplopia	1	1.06
Bell 's palsy right	1	1.06
Change in behavior	1	1.06

*SOL* Space Occupying Lesion, *CVA* Cerebrovascular accident.

The mean Height (H) (± standard deviation) of the retrobulbar bony pyramid in mm (measurement of imaginary/ determined line from point A to point B (Fig. 1A)) was 23.9 ± 2.9.

The V1–V5 areas are shown in Fig. 1B.

The mean V1—total retrobulbar orbital volume was 5956 ± 1568 mm^3^. The mean V2—measured the area between P2 and the optic foramen was 3442 ± 1028mm^3^. The mean V3—measured the area between P3 and the optic foramen was 1510 ± 520mm^3^. The mean V4—measured the area between P4 and the optic foramen was 470 ± 159mm^3^. The mean V5—measured the area between P5 and the optic foramen was 88 ± 34 mm^3^ Table 3.

**Table 3:** Mean volume of orbital retrobulbar segments.

The mean volume	
V1	5956 ± 568 mm^3^
V2	3442 ± 1028mm^3^
V3	1510 ± 520mm^3^
V4	470 ± 159mm^3^
V5	88 ± 34mm^3^

This table demonstrates the measured volume of each segment V1–V5 of the orbital retrobulbar space.

The subtractions of differences in orbital volumes are shown graphically in (Fig. 2).

**Figure 3:**
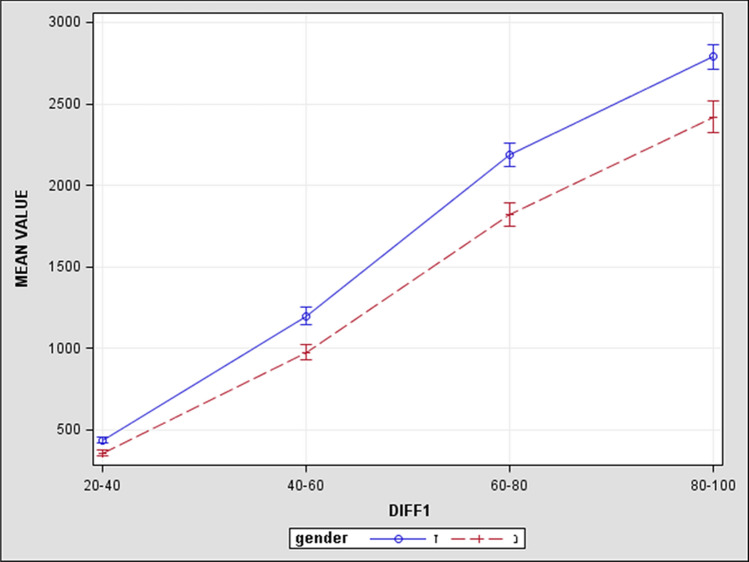
The blue line represents the results of male patients and red dashed line represents results of female patients. The X axis—represents points of calculated differences between the retrobulbar orbital volumes, they are shown at equal distances from each other. The Y axis—represents subtraction differences in the nearest retrobulbar orbital volumes in mm^3^. The numeric value of subtraction differences of volumes being smaller in females we find the same slope as in males and the progression of decline is most obvious between V80%-V60% and V60%-V40% in both genders, permitting us to arrive at conclusions equally for both genders.

**Figure 4:**
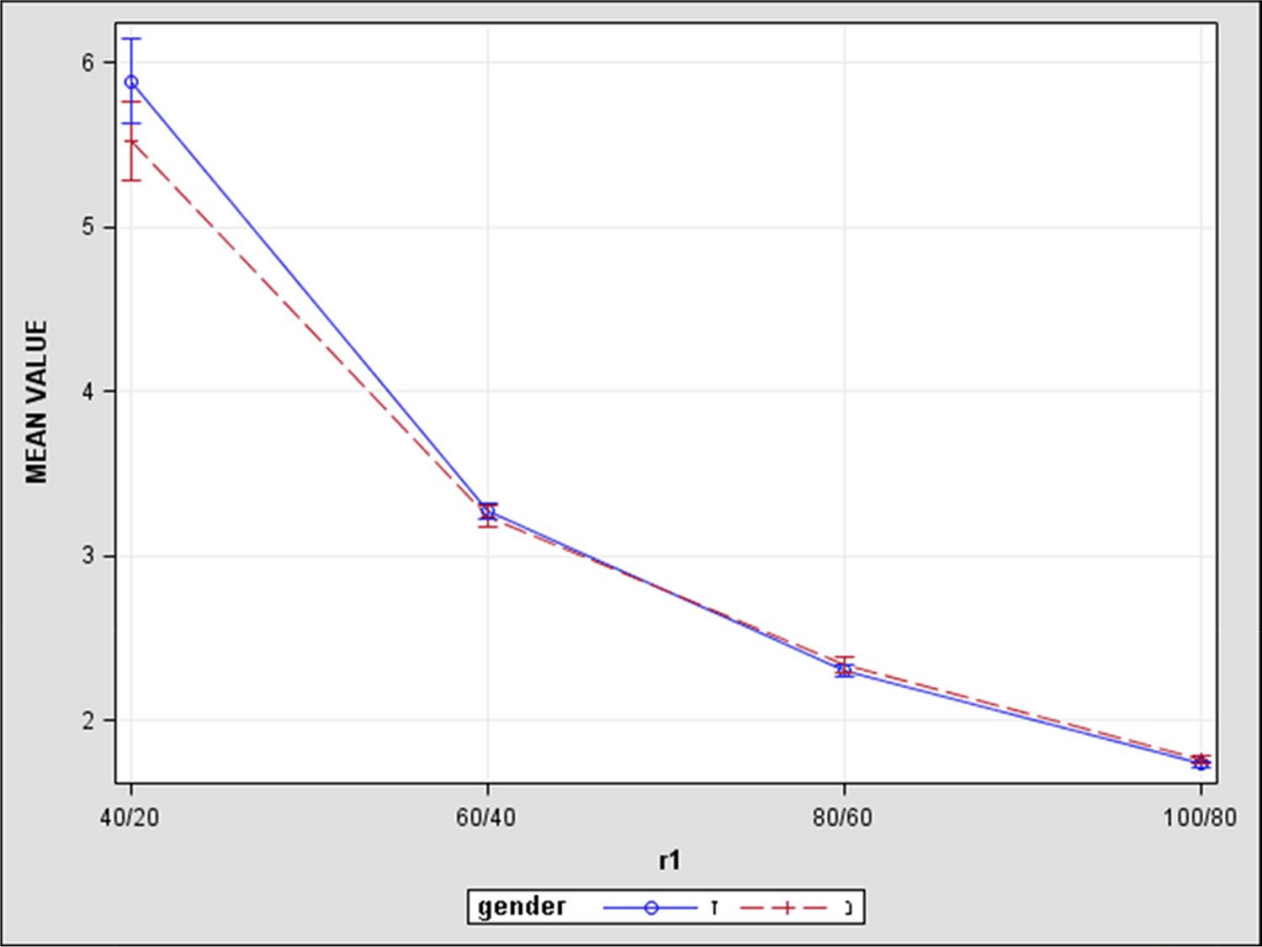
Blue line represents results of male patients and red dashed line represents results of female patients. The X axis—represents points of calculated differences between retrobulbar orbital volumes, and they are shown at equal distances from each other. The Y axis represents ratio differences in nearest retrobulbar orbital volumes. The numeric value of ratio differences of volumes is a little smaller in females but they still have the same slope as in males, permitting us to come to a conclusion of gender equality.

Although the numeric value of ratio differences of volumes is a little smaller in females, we found the same progression of decline in volume in both genders (Figs. 3 and 4).

## Discussion

The concept of the orbital surgical apex is an important one and a well-accepted concept among orbital surgeons^11,14^. The apex has never been defined although it is widely understood as the area where a significant decline in volume occurs, leading to the increased crowding of crucial structures. Surgical problems involving this area are more complex and need special attention.

Neuroradiologists although aware of the importance of orbital volume, are not generally familiar with the “apex” concept as the area has not been defined specifically^15–22^. They would however be aware of its significance if the location could be defined. Thus, if aware of it they could potentially communicate and alert the ophthalmologist to a surgical problem earlier, i.e. risk of optic nerve compression caused by sub-periostial abscess involving the defined surgical apex, or a tumor biopsy in the surgical apex^23,24^ Consequently, surgeons would be better able to accurately prepare and pre-plan the surgical approach^25,26^.

Our search in “PubMed” with keywords “surgical orbital apex/orbital apex”, found no publications in the ophthalmological or radiological literature. To the best of our knowledge this is the first study of its kind to standardize CT scan data of the retrobulbar space. We did find however, multiple references on measurements in orbit, orbit volume reconstruction, and literature concerned with decompression of the optic nerve and apex^5^ Although some papers disscussed orbital apex, it was never defined specifically.

Choosing the anatomical landmarks was a challenge as these are variable in different patients and are not always simple to identify on CT^27^. It was therefore decided to use the bony walls of the orbit and the length of optic nerve from its insertion in the globe to its exit from the orbit into the optic canal as the anatomical landmarks for our study. These landmarks were ideal as they are readily recognized on CT and their data easily standardized in patients of all ages, genders and ethnicity^17,28,29^ The exit point of the optic nerve from the orbit at the optic foramen was determined in an axial slice at a height where the anterior clinoid processes could be identified as a landmark.

In the current study, data from 100 orbital CT scans of randomly selected patients was processed and analyzed.

Our results demonstrated that in the area of the V3 retrobulbar volume (the posterior 3/5 of the orbital volume) the decline in volume more than doubles and is statistically significant. Thus, in this posterior area the crowding of structures like nerves, blood vessels and muscles is greater. However, the ratio difference at V4 to V5 is nearly six-fold which is statistically significant. These results led us to make two separate definitions regarding the orbital surgical apex.

We defined the posterior 3/5 of retrobulbar space where the volume decline more than doubles, was the orbital surgical apex. However, the posterior 1/5, in which the volume decline more than fivefold is defined by usas the absolute orbital apex. The posterior fifth is where we find a mass on occasion such as a hemangioma which does not necessarily cause proptosis but can cause significant optic nerve compromise. These lesions are extremely difficult to remove and the clinical situation of masses in this area is different and even more complex. Therefore, defining the absolute apex in addition to the surgical apex is probably very important and with clinical significance.

There was only a small number of children under the age of 10 in accordance with practice policy of avoiding CT due to the risks of radiation in this age group. It may be worthwhile to examine the data of more patients in this age group in order to compare results with the adult population.

This is the first study to define the surgical apex and we believe that it will provide a practical neuro-radiological tool allowing the physician to identify high risk cases such as sub-periosteal abscess. We believe that this study will increase awareness among both ophthalmologists, neuroradiologists, and other physicians as to the concept of the surgical apex. In cases of sub-periosteal abscess, it should potentially be of immense help in diagnosing the cases at higher risk of developing optic nerve compression. This definition could possibly be used as an extra clinical tool to identify those patients needing urgent surgery. However, it should be emphasized that the decision to operate is based on clinical findings^30^. Likewise posterior masses lying in the surgical apex or absolute apex can be easily classified by location and considered carefully before surgery. The definition should also increase the awareness regarding the area needing surgical decompression in Thyroid Orbitopathy where the optic nerve is compromised^14^. A further study will need to be carried out to examine the effectivity of this definition in clinically relevant cases.

Posterior masses, lying in the surgical apex or absolute apex can be easily classified by location using this concept and considered carefully before surgery.

A further study will need to be carried out to examine the application of this definition in abnormal orbits and with regard to clinical presentation. In addition, the surgical orbital apex in the under-ten age group and in different ethnicities will need to be examined and determined.

## Research ethics approval: human participants

The Emek Medical Center Ethics Committee review board approved the study number: 0045-11EMC.

The Ethics committee review board at Emek Medical Center, Israel, is organized and operates according to ICH-GCP guidelines and to the applicable laws and regulations. Emek Medical Center Ethics Committee review board.
